# Petroclival Clinoidal Folds and Arachnoidal Membranes of the Anteromedial Incisural Space: Clinical Anatomy for Neuro Critical Care

**DOI:** 10.3390/diagnostics13203203

**Published:** 2023-10-13

**Authors:** Lorenzo Pescatori, Samanta Taurone, Antonello Ciccarelli, Mauro Palmieri, Alessandra Serraino, Marco Artico, Francesco Fornai, Yaroslava Longhitano, Christian Zanza, Manfredi Tesauro, Gabriele Savioli, Selenia Miglietta, Pasqualino Ciappetta

**Affiliations:** 1 Department of Neurosurgery, S. Eugenio Hospital of Rome, 00144 Rome, Italy; 2 Department of Movement, Human and Health Sciences, Division of Health Sciences, University of Rome “Foro Italico”, 00135 Rome, Italy; 3 Human Neurosciences Department, A.U.O. “Policlinico Umberto I” Neurosurgery Division, Sapienza University, 00185 Rome, Italyalessandra.serraino@uniroma1.it (A.S.); 4 Department of Sensory Organs, “Sapienza” University of Rome, 00185 Rome, Italy; marco.artico@uniroma1.it; 5 IRCCS Neuromed, 86077 Pozzilli, Italy; francesco.fornai@unipi.it; 6 Department of Translational Research and New Technologies in Medicine and Surgery, University of Pisa, 56126 Pisa, Italy; 7 Department of Anesthesiology and Perioperative Medicine, University of Pittsburgh, Pittsburgh, PA 15260, USA; 8 Department of Emergency Medicine, Humanitas University Hospital, 20089 Rozzano, Italy; 9 Italian Society of Prehospital Emergency Medicine (SIS 118), 74121 Taranto, Italy; 10 Post Graduate School of Geriatric Medicine, University of Rome “Tor Vergata”, 00133 Rome, Italy; 11 Department of Systems Medicine, University of Rome “Tor Vergata”, 00133 Rome, Italy; 12 Emergency Department, IRCCS Fondazione Policlinico San Matteo, 27100 Pavia, Italy; gabrielesavioli@gmail.com; 13 Department of Anatomical, Histological, Forensic Medicine and Orthopedics Sciences, “Sapienza” University of Rome, 00185 Rome, Italy; selenia.miglietta@uniroma1.it; 14 Catholic University of Sacred Heart, 00168 Rome, Italy

**Keywords:** clinoid processes, petroclival folds, oculomotor triangle, anterior petroclinoid ligament, posterior petroclinoid ligament, interclinoid ligament, neuroradiology, emergency medicine, neuroanesthesia

## Abstract

A systematic and narrative literature review was performed, focusing attention on the anatomy of the area located at the junction of the sphenoid and the basal portion of the temporal bone (petrous bone, petrous apex, upper petro-clival region) encircled by the free edge of the tentorium, the insertion of the tentorium itself to the petrous apex and the anterior and posterior clinoid processes that give rise to three distinct dural folds or ligaments: the anterior petroclinoid ligament, the posterior petroclinoid ligament and the interclinoid ligament. These dural folds constitute the posterior portion of the roof of the cavernous sinus denominated “the oculomotor triangle”. The main purpose of this review study was to describe this anatomical region, particularly in the light of the relationships between the anterior margin of the free edge of the tentorium and the above-mentioned components of the sphenoid and petrous bone.

## 1. Introduction

The anterior and middle incisural spaces are regions of exceptional anatomical and neurosurgical interest and are characterized by complex relationships between the bony, dural, arachnoid and neurovascular structures [1,2,3]. The anatomical relationships in the anterior incisural space and those in the posterior incisural space are extremely complex, especially as far as the vascular component is concerned, because the anterior incisural space contains all the components of the circle of Willis and the bifurcation of the internal carotid and basilar arteries, while the posterior incisural space contains the convergence of the internal and basal cerebral veins and several of their tributaries to the vein of Galen [4].

In this area, comprising petrous bone, petrous apex, upper petro-clival region and encircled by the free edge of the tentorium, the insertion of the tentorium itself to the petrous apex and to the anterior and posterior clinoid processes gives rise to three distinct dural folds or ligaments: the anterior petroclinoid ligament, the posterior petroclinoid ligament and the interclinoid ligament [5,6,7,8,9,10,11]. These dural folds constitute the posterior portion of the roof of the cavernous sinus denominated “the oculomotor triangle” [1,2,3,12,13].

The main purpose of this review study is to describe this anatomical region, paying particular attention to the relationships between the anterior margin of the free edge of the tentorium and the above-mentioned components of the sphenoid and petrous bone. Moreover, the authors aim was to examine the relationships between these components and the arachnoid membranes of the basal cisterns belonging to the anterior and middle incisural spaces.

For this purpose, anatomical dissections on fresh (not more than 48 h postmortem) specimens which had not been injected or fixed in formalin were performed. The possibility of dissecting fresh cadavers avoided any morphologic alterations of the arachnoid membranes caused by the formalin-fixation process [14,15,16].

Formalin-fixed injected specimens were used occasionally in this study for the description of specific anatomical details. In addition, some of the photographs presented in this study were obtained in vivo during neurosurgical procedures involving this region.

This study demonstrates how anatomic knowledge of this region is important in neurosurgical practice, particularly for the surgical treatment of pathologies located in the anterior and middle skull base.

## 2. Materials and Methods

### Ethics Statement and Patients

The experimental protocol was approved by the local ethics committee and strictly adhered to the guidelines of the Declaration of Helsinki for research on human participants and in agreement with the ARVO declaration for use of human samples in ophthalmic and vision research.

Eight fresh adult cadaveric heads were analyzed in this study. All the dissections and photographs were performed before 2006, when the ethic committee’s permission was not requested. The cranial vault was removed circumferentially in order to expose the entire skull base. The brains were left in place. This allowed a better visualization and comprehension of relationships between arachnoid membranes of the central skull base, the sellar region and the anterior and lateral incisural spaces.

The anatomical study was focused on the description of the relationships between bony (anterior clinoid process, posterior clinoid process, optic canal, optic strut, superior orbital fissure), dural (anterior and posterior petroclinoid ligament, interclinoid ligament, proximal and distal dural ring, carotid-oculomotor membrane, falciform ligament, diaphragma sellae, carotid collar), arachnoid, Liliequist’s membrane, peri-mesencephalic cisterns of the anterior and middle incisural space) and neurovascular (optic nerve, oculomotor nerves, internal carotid artery) structures.

A CANON 1Ds MarkIII camera fitted with a MacroLens 100 mm or MP-E 65 1-5X in order to obtain a reproduction ratio of 1:1 or more (2:1–3:1) was used. The study was accomplished using an operating microscope (Carl Zeiss Corp., Oberkochen, Germany)

## 3. Results

### 3.1. Anterior and Posterior Clinoid Process

**Figure 1:**
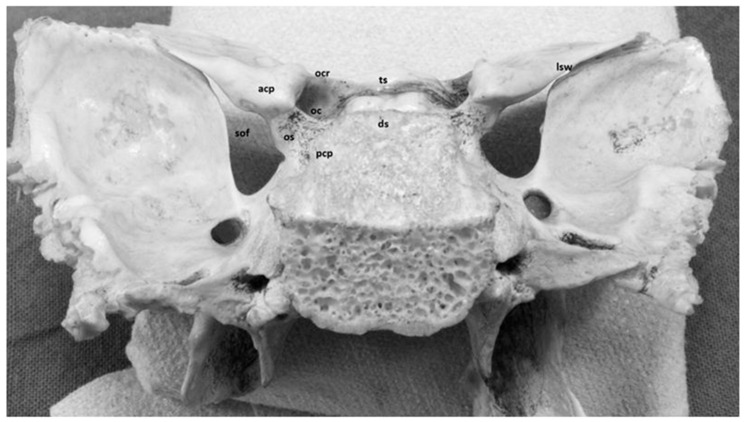
Sphenoid bone, postero-superior view. The anterior clinoid process (*acp*) is the bony prominence localized at the medial limit of the lesser sphenoid wing (*lsw*). It represents the bony component of both the superior orbital fissure (*sof*) and anterior portion of the roof of the cavernous sinus. The connection of the anterior clinoid to the skull is characterized by three main sites of attachment: the lesser sphenoid wing laterally, the roof of the optic canal (*ocr*) and the planum sphenoidale medially and the optic strut (*os*)infero-medially.

**Figure 2:**
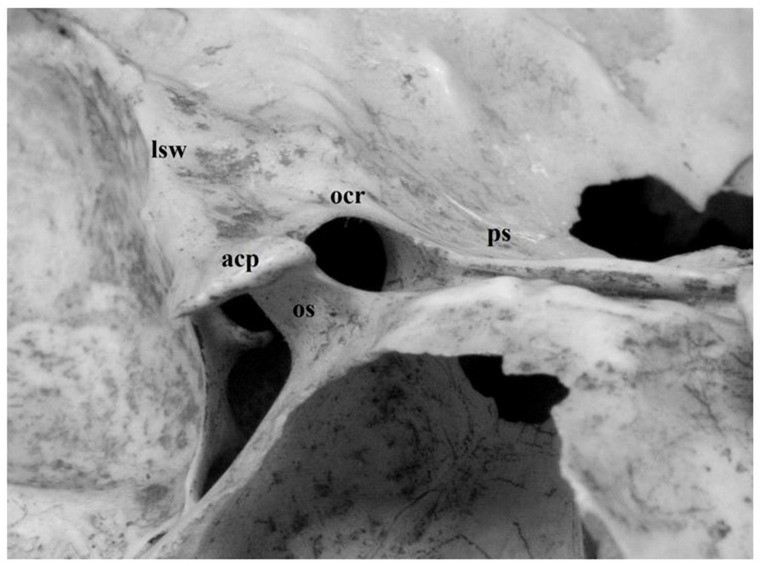
Anterior clinoid process (*acp*), magnified view. In this anatomic preparation, attachment points of the anterior clinoid to the skull can be clearly observed: lesser sphenoid wing laterally (*lsw*), optic canal roof (*ocr*) and planum sphenoidale (*ps*) anteromedially, optic strut (*os*)inferomedially. The optic strut is also the main constituent of the floor of the optic canal.

The minor wings of the sphenoid bone present the anterior clinoid process, which are smooth and have an interface for the junction with the frontal bone anteriorly. The most interesting features of these small wings are the optic holes that allow the passage of the optic nerves. They represent the bony component of both the superior orbital fissure and the anterior portion of the roof of the cavernous sinus (Figure 1 and Figure 2). Knowledge of their morphological characteristics is essential to avoid iatrogenic lesions of anatomical structures such as the optic nerve and the internal carotid artery.

The connection of the anterior clinoid to the skull is characterized by three main sites of attachment: the lesser sphenoid wing laterally, the roof of the optic canal, the planum sphenoidale medially and the optic strut infero-medially. The optic strut extends from the inferomedial margin of the anterior clinoid process to the body of the sphenoid bone, separating the superior orbital fissure from the optic canal and representing the lateral portion of the floor of the optic canal (Figure 2).

The posterior clinoid processes represent the postero-lateral appendix of the dorsum sellae (Figure 1).

Both these structures are derived from duplication of the anterior margin of the free edge of the tentorium at the petrous apex.

### 3.2. Dural Relationships and Dural Folds

**Figure 3:**
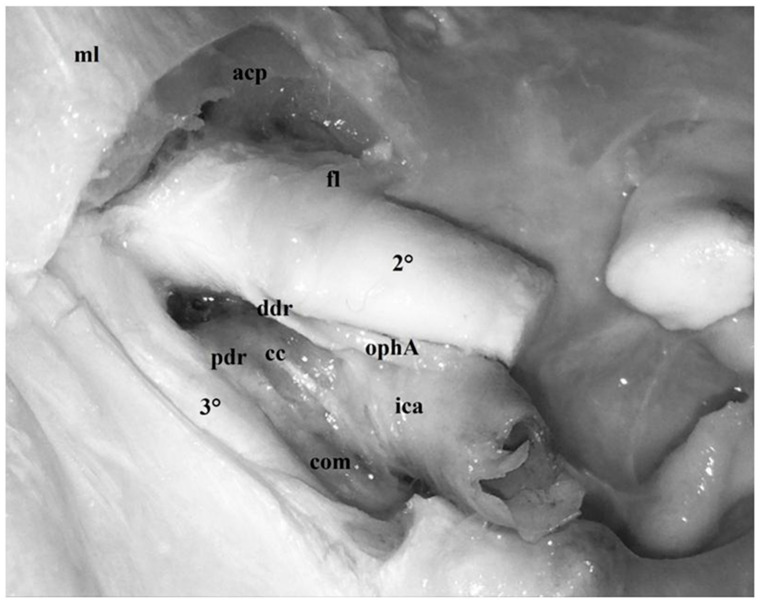
Formalin-fixed, injected specimens, postero-superior view, left side. The left anterior clinoid process (*acp*) has been removed through an intradural clinoidectomy and the periosteal layer of dura mater localized below the clinoid has been exposed. Overall, the triangular area constituted by dura mater between the optic nerve and the oculomotor nerve is called carotid triangle and represents the deeper layer of the anterior half of the roof of the cavernous sinus (the first two layers are the meningeal dura *md* and the bony component of the anterior clinoid process). The denomination of the meningeal dura in this region is variable and depends on the localization of the membrane itself. Between the oculomotor nerve and the ICA (*ica*), it forms the carotid-oculomotor membrane (*com*) separating the oculomotor nerve from the ICA. At the exit point of the ICA from the cavernous sinus at the anterior portion of the carotid triangle, the carotid-oculomotor membrane encircles the ICA itself constituting the proximal dural ring (*pdr*), which represents the inferior limit of the clinoid segment. The same layer of dura mater accompanies the clinoid portion of the ICA as carotid collar (*cc*) (*fl*: falciform ligament; *2°*: optic nerve; *3°*: oculomotor nerve).

**Figure 4:**
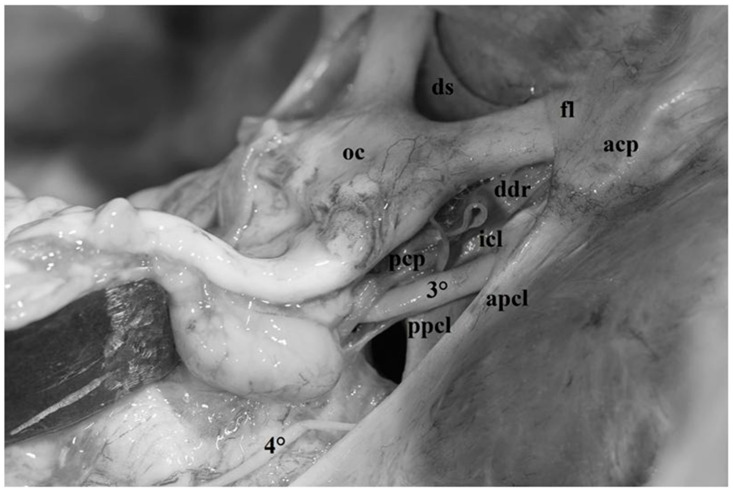
Fresh, non-formalin-fixed specimen, anatomic dissection, right side. In this photograph, the complex anatomic organization of the meningeal layer of the dura mater around the anterior clinoid process can be completely understood. The dura propria cover the superior surface of the anterior clinoid process (*acp*). Anterolaterally, this dural layer is continuous with the falciform ligaments (*fl*) which represents the postero-lateral portion of the roof of the optic canal. Medially, together with the basal arachnoid membrane, the dura propria continues as diaphragma sellae(*ds*) extending until the clivus, whereas postero-medially, it constitutes the distal dural ring (*ddr*) embracing the internal carotid artery (ICA) and represents the superior limit of the clinoid segment of the ICA itself. In this dissection, the dural folds constituting the oculomotor triangle are evident: anterior petroclinoid ligament (*apcl*), posterior petroclinoid ligament (*ppcl*), interclinoid ligament (*icl*). Note how the oculomotor nerve (*3°*) penetrates in the central part of the oculomotor triangle. Posteriorly, the trochlear nerve (*4°*) pierces the tentorium.

The anterior clinoid process, together with its dural attachments, represents the anterior portion of the roof of the cavernous sinus. Superiorly, the clinoid process is covered by a thick layer of dura mater also called meningeal layer or dura propria. Anterolaterally, this layer is continuous with the falciform ligaments, which represent the postero-lateral portion of the roof of the optic canal. Medially, the dura propria proceeds as the diaphragma sellae, extending as far as the clivus, whereas postero-medially, it constitutes the distal dural ring, embracing the internal carotid artery (ICA) and representing the superior limit of the clinoid segment of the ICA itself (Figure 3 and Figure 4).

Intradural removal of the anterior clinoid process obtained by disinserting its three points of attachment to the skull base (lesser sphenoid wing, orbital roof and planum sphenoidale) reveals a second, deeper layer of dura mater also known as the periosteal layer or reticular layer which covers the inferior surface of the anterior clinoid. Situated between the optic nerve and the third cranial nerve, this dural layer constitutes the carotid triangle, which represents the deepest layer of the anterior half of the cavernous sinus (Figure 3).

Between the oculomotor nerve and the ICA, this layer forms the carotid-oculomotor membrane, thus separating the oculomotor nerve from the ICA. On the exit point of the ICA from the cavernous sinus in the anterior portion of the carotid triangle, the carotid-oculomotor membrane encircles the ICA itself, thus constituting the proximal dural ring which represents the inferior limit of the clinoid segment. This layer of dura mater accompanies the clinoid portion of the ICA as a carotid collar (Figure 3). On the medial side, the dural collar may be easily dissected from the wall of the ICA owing to the presence of a pouch which was denominated carotid cave by Kobayashi et al. [8].

Posteriorly, the reticular layer of the carotid-oculomotor membrane and the meningeal layer of the distal dural ring merge to constitute a single layer of dura, which covers the triangular space delimited by the petrous apex and the anterior and posterior clinoid processes.

### 3.3. Free Edge of the Tentorium and Incisural Spaces

The term free edge of the tentorium is conventionally used to indicate the margin of the tentorium not attached to the skull and delimits the incisural space on the medial side.

**Figure 5:**
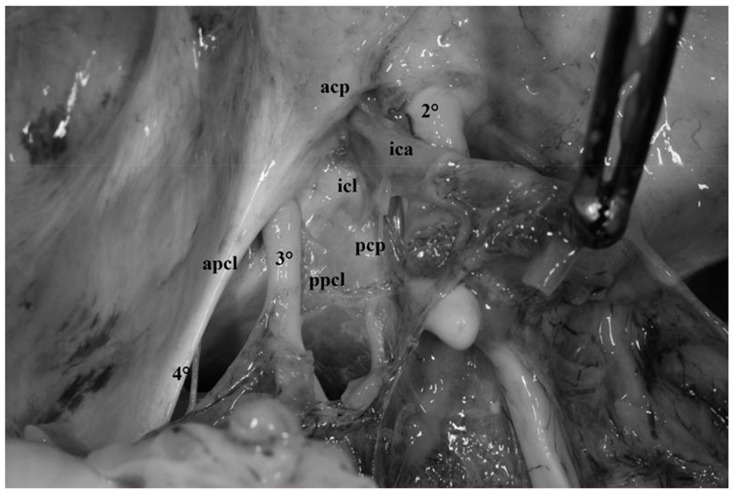
Fresh, non-formalin-fixed specimen, anatomic dissection, left side. In this dissection, the dural folds forming the oculomotor triangle can be clearly observed. The first two components are the anterior and posterior petroclinoid ligaments (*apcl*, *ppcl)* coursing between the petrous apex and the anterior and posterior clinoid process, respectively (*acp*, *pcp*). The third component is represented by the interclinoid ligament (*icl*) localized between the anterior and posterior clinoids. In this dissection, the cisternal portion and the petroclinoid portion of the oculomotor nerve can be clearly visualized (see text for details). Note how the third nerve (*3°*) penetrates the dura in the central part of the oculomotor, whereas the trochlear nerve (*4°*) enters the dura at the postero-lateral edge of this triangle.

Anteriorly, it adheres to the petrous apex and splits into two distinct components, also known as dural folds or ligaments (Figure 4 and Figure 5).

The first component is attached to the anterior clinoid and is called anterior petroclinoid ligament; the second component is attached to the posterior clinoid and is known as the posterior petroclinoid ligament. Moreover, between the anterior and posterior clinoid, the dura of the skull base depicts a clearly distinguishable dural fold called the interclinoid ligament (Figure 4 and Figure 5).

**Figure 6:**
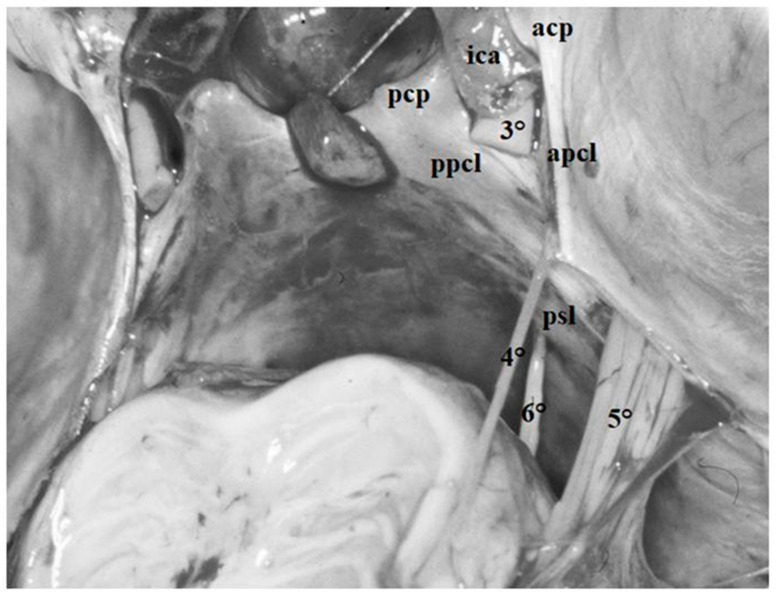
Fresh non-formalin-fixed specimens, anatomic dissection. The arachnoid trabeculae of the mesencephalic portion of the Lilequist’s membrane were removed and the oculomotor nerves (*3°*) were transected to show the space between the upper-middle clivus and the brainstem within the posterior half of the anterior incisural space. On the right side, below the cisternal portion of the fourth (*4°*) and the fifth (*5°*) cranial nerves, the sixth cranial nerve (*6°*) exits the brainstem at the pontomedullary sulcus and ascends within the prepontine cisternto piercing the dura of the clivus and eventually enters within the Dorello’s canal. The roof of the canal is constituted by the petrous sphenoid ligament (aka Gruber’s ligament, *psl*) running between the petrous apex and the dorsum sellae just below the posterior clinoid process (*pcp*). Further structures observable in this dissection are the anterior clinoid process (*acp*), the anterior petroclinoid ligament (*apcl*) and the ICA (*ica*).

This dural fold delimits a triangular space pierced by the oculomotor nerve, which is commonly called the “oculomotor triangle” [12,16,17,18,19,20,21,22,23,24] (Figure 4 and Figure 5). It represents the posterior component of the roof of the cavernous sinus through which the oculomotor and trochlear nerves enter the cavernous sinus. The oculomotor nerve penetrates the dura in the central part of this triangle, the oculomotor triangle, and the trochlear nerve enters the dura at the postero-lateral edge of this triangle (Figure 4, Figure 5 and Figure 6). The Gruber ligament or petrosphenoid ligament (PSL) passes between the leaves of the posterior petroclinoid fold from the petrous apex to the lateral border of the dorsum sellae, just below the posterior clinoid process (Figure 6).

**Figure 7:**
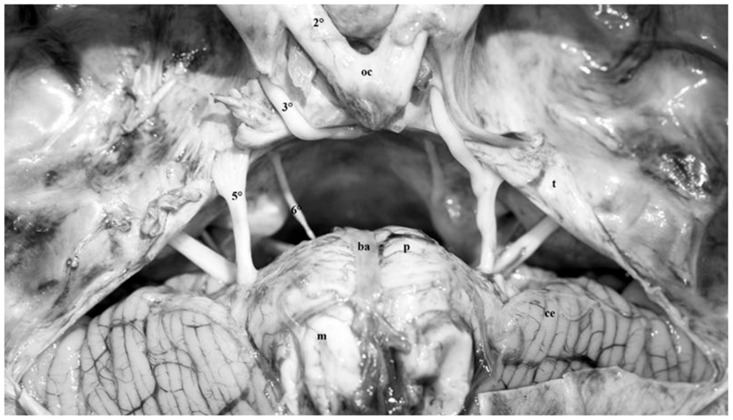
Fresh non-formalin-fixed specimen, anatomic dissection, superior view of the skull base. The cerebral hemispheres were removed and the tentorium (*t*) was transected in a medio-lateral direction from the tentorial edge to show the anterior and middle incisural spaces and their content. The anterior incisural space is the part of incisural space localized ventral to the brain stem. Postero-inferiorly, it spreads between the brainstem and the clivus; Anteriorly, it encircles the optic chiasm. Above the optic chiasm, it reaches the subcallosal area. Below the chiasm and the third ventricular floor, it extends backward until it reaches the interpeduncular fossa and cistern. The middle incisual space is lateral to the brain stem (*m*: mesencephalon, *p*: pons; *ba*: basilar artery; *ce*: cerebellar hemisphere; *5°*: trigeminal nerve; *6°*: abducens nerve; *3°*: oculomotor nerve; *2°*: optic nerve; *oc*: optic chiasm).

As mentioned above, the free edge of tentorium represents the lateral boundary of the tentorial incisura. Consequently, the tentorial incisura may be defined as the anatomical region located between the free edge of the tentorium and the upper brainstem (Figure 6 and Figure 7). It represents the only point of communication between the supratentorial and infratentorial spaces and is subdivided into three different portions: anterior, middle and posterior incisural space.

The anterior incisural space is located anteriorly to the brainstem, the middle incisural space is lateral, whereas the posterior incisural space is posterior (Figure 6 and Figure 7).

The incisural space is occupied by the mesencephalon, the pons and, partially, by the superior surface of the cerebellar hemispheres.

### 3.4. Anterior Incisural Space

The anterior incisural space is the part of incisural space located ventrally to the brainstem. Postero-inferiorly, it spreads between the brainstem and the clivus. Anteriorly, it encircles the optic chiasm. Above the optic chiasm, it reaches the subcallosal area. Below the chiasm and the floor of the third ventricle, it extends backward until it reaches the interpeduncular fossa and cistern. Laterally, it opens into the Sylvian fissure, whereas posteriorly it opens into the middle incisural space between the brainstem and the uncus (Figure 6 and Figure 7).

### 3.5. Middle Incisural Space

The middle incisural space is located laterally to the brainstem. Above the free edge of the tentorium, this space extends between the mesial temporal lobe and the midbrain, whereas below the tentorium it continues between the upper pons and the cerebellum. The cisternal spaces related to this space are the crural cistern, anteriorly, and the ambient cistern posteriorly (Figure 6 and Figure 7).

### 3.6. Arachnoid and Cisternal Relationships

The cisternal spaces related to the anterior incisural space are the carotid cistern, the chiasmatic cistern, the pituitary stalk cistern, the lamina terminalis cistern and the interpeduncular cistern. The medial carotid membrane separates the carotid cistern from the chiasmatic cistern. Given their importance from both anatomical and surgical points of view, these membranes will be discussed separately together with the organization of arachnoid membranes located near the pituitary stalk in specific paragraphs.

The cisternal spaces related to the middle incisural space are the crural cistern and the ambient cistern.

### 3.7. Medial Carotid Membrane

**Figure 8:**
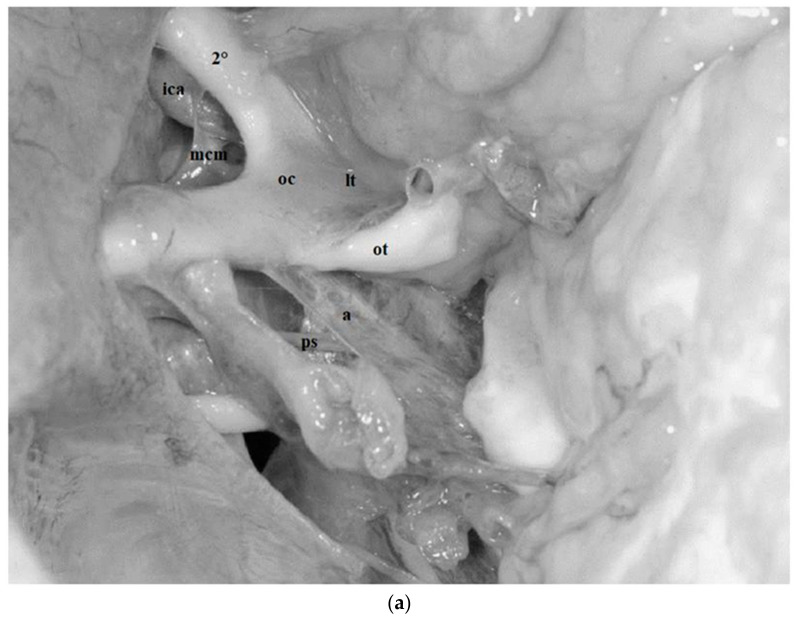

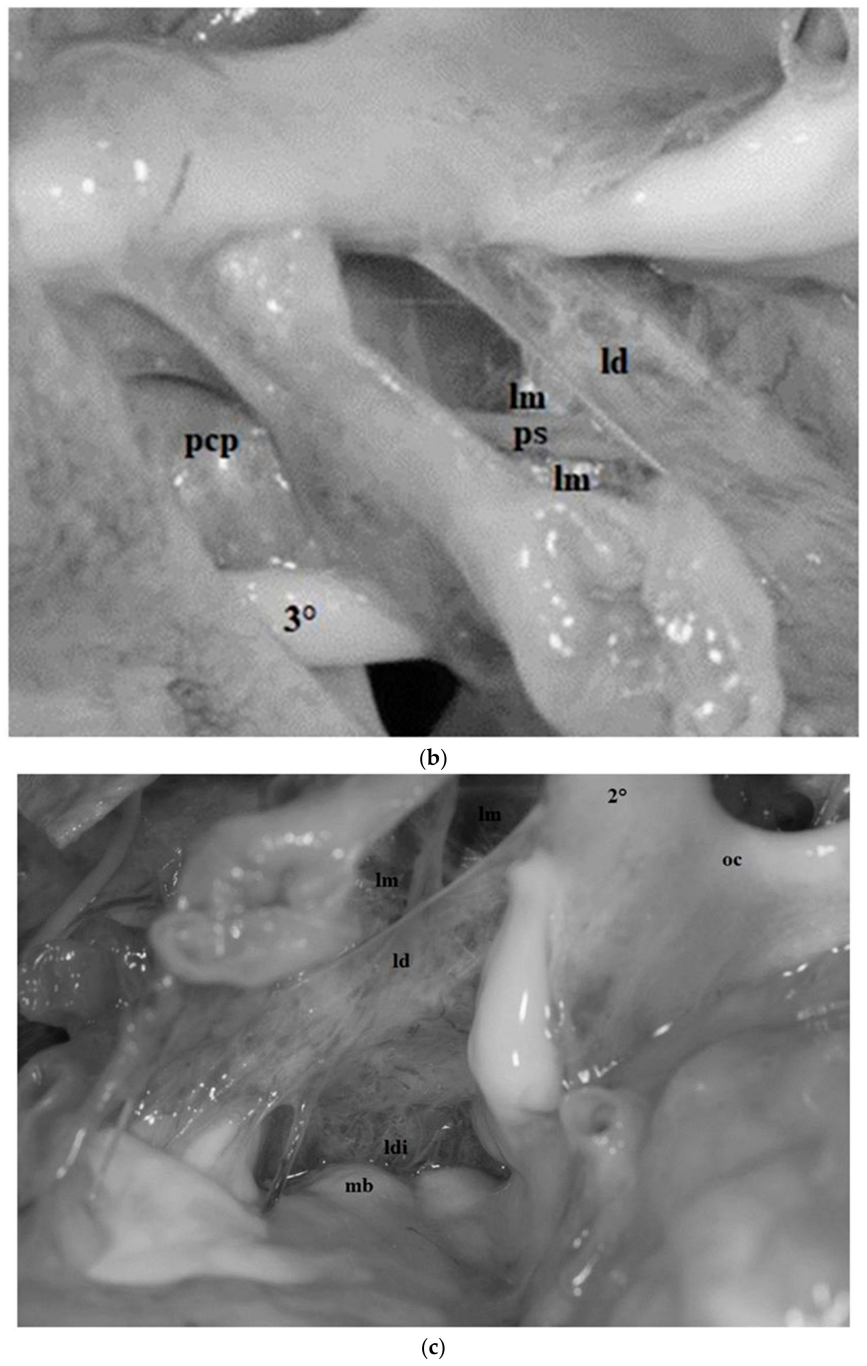
(**a**) Fresh, non-formalin-fixed specimen, anatomic dissection simulating a left fronto-temporal trans-Sylvian approach. Some of the arachnoid membranes of the anterior space were exposed. The medial carotid membrane (*mcm*) origin from the inferomedial side of the supraclinoid ICA (*ica*) and attaches on the inferolateral surface of the optic chiasm (*oc*) reflecting over the anterolateral surface of the pituitary stalk (*ps*). It separates the carotid from the chiasmatic cistern. Above the optic chiasm the lamina terminalis (*lt*) is visible. Posteriorly and inferiorly arachnoid trabeculae belonging to the basal arachnoid and to the diencephalic portion of the Lilequist’s membrane are visible (*a*). (*ot*: optic tract). (**b**) Fresh, non-formalin-fixed specimen, anatomic dissection simulating a left fronto-temporal trans-Sylvian approach, enlarged view. The components of the Lilequist’s membrane can be identified. The diencephalic portion (*ld*) runs from the dorsum sellae to the mammillary bodies whereas the mesencephalic portion (*lm*) extends from the dorsum sellae to the ponto-mesencephalic sulcus. (*3°*: oculomotor nerve; *pcp*: posterior clinoid process; *ps*: pituitary stalk). (**c**) Fresh, non-formalin-fixed specimen, anatomic dissection, left side. The frontal lobe has been spatulated, through a more frontal trajectory the diencephalic portion of the Lilequist’s membrane (*ld*) can be clearly observed and followed until its attachment (*ldi*) to the mammillary bodies (*mb*). Below the diencephalic component the mesencephalic portion (*lm*) separating the interpeduncular from the pre-pontine cistern is visible. (*oc*: optic chiasm; *2°*: optic nerve).

**Figure 9:**
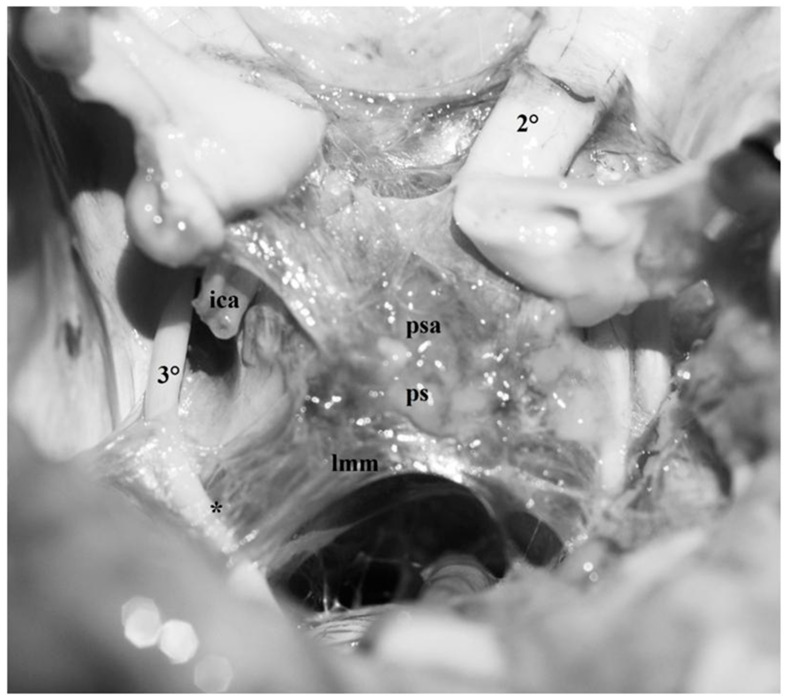
Fresh, non-formalin-fixed specimen, anatomic dissection, superior view. The frontal and temporal lobes were removed leaving the basal arachnoid membrane in place and the optic chiasm was cut. In this dissection, the anterior incisural space and the arachnoid membranes of the region can be clearly visualized. The pituitary stalk (*ps*) is approximately localized in the central part of the anterior incisural space. Anteriorly, it is covered by arachnoid trabeculae (*psa*) originating from the medial carotid membrane and from the basal arachnoid membrane of the frontal lobes. Posteriorly, the mesencephalic portion of the Lilequist’s membrane (*lmm*) runs from the dorsum sellae to the pontomesencephalic sulcus, separating incompletely the pre-pontine from the interpeduncular cistern. Ventral trabeculae of the Lilequist’s membrane cover the posterior surface of the pituitary stalk completing, together with the above-mentioned arachnoid membranes, the funnel shaped arachnoid collar delimiting the pituitary stalk cisternal space. Postero-laterally, note the wonderful reflection of the Lilequist’s membrane over the oculomotor nerve (*).

The medial carotid membrane binds to the optic chiasm and to the surface of the infundibulum, strengthening the arachnoid collar formed by the basal arachnoid membrane and dividing the chiasmatic cistern from the carotid cistern (Figure 8a and Figure 9).

### 3.8. The Membrane of Liliequist

**Figure 10:**
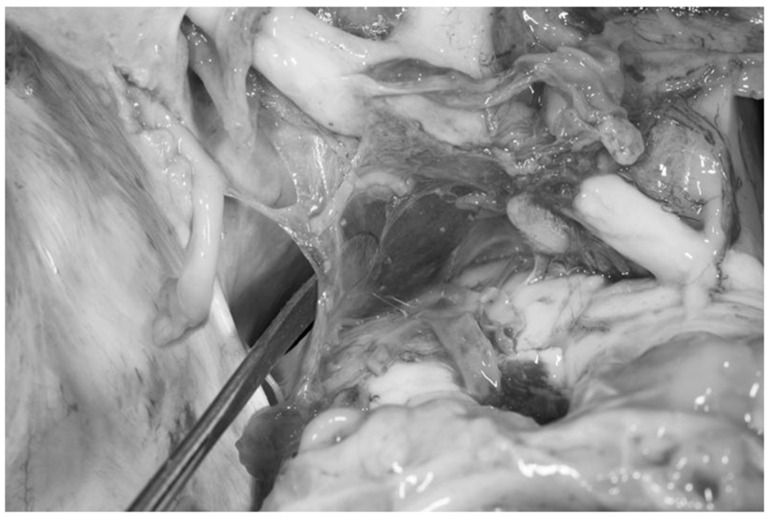
Fresh non-formalin-fixed specimen, superolateral view, left side. The dissector has been placed below the mesencephalic portion of the Lilequist’s membrane to demonstrate how it separates the prepontine cistern from the interpeduncular one.

This membrane arises from the arachnoid membrane located at the posterior clinoid processes and the dorsum sellae. It then continues, spreading superiorly and caudally, and subsequently divides to form two diaphragms: the diencephalic and the mesencephalic layers [25]. The diencephalic layer projects upwards and backwards by binding to the mammillary bodies, hermetically separating the chiasmatic and the interpeduncular cisterns [25]. The lateral border of the midbrain and diencephalic membranes extends into the arachnoid membrane surrounding the oculomotor nerves. It has been observed that the rod-shaped structures of fibrous tissue originating from the superior surface of Liliequist’s membrane adhere to the aspect of the optic chiasm and to the postero-lateral and posterior surface of the pituitary stalk, overlaying to the basal arachnoid membrane (Figure 8, Figure 9 and Figure 10).

### 3.9. Pituitary Stalk and Pituitary Stalk Cisternal Space

The pituitary stalk connects the pituitary gland to the floor of the third ventricle. This connection is made through the anterior incisural space entering the opening of the diaphragma sellae. The pituitary stalk is located in the chiasmatic cistern. A small distal portion of pituitary stalk adjacent to the diaphragma sellae is extra-arachnoidal [26,27,28]. The arachnoid basal membrane extends around the stalk and reflects upwards on its plane at the site of penetration on the diaphragma sellae (Figure 8, Figure 9 and Figure 10).

The anterolateral and postero-lateral surfaces are surrounded by folds arising from the medial carotid membrane. These components form a “cone-shaped” arachnoid ring around the pituitary peduncle demarcating a separate cisternal space, simultaneously surrounded by the chiasmatic cistern (Figure 8, Figure 9 and Figure 10).

### 3.10. Cranial Nerves

The cranial nerves related to the anterior and middle incisural space are the optic nerve, oculomotor nerve, trochlear nerve, trigeminal nerve and abducens nerve.

The anatomy of the third, fourth, fifth and sixth cranial nerves will be discussed in detail.

### 3.11. Oculomotor Nerve

The oculomotor nerve may be subdivided into to four distinct segments: cisternal, petroclinoid, trigonal and cavernous.

The first segment extends from the origin of the nerve in the interpeduncular cistern, between the posterior cerebral artery and the superior cerebellar artery, to the posterior petroclinoid ligament (Figure 4 and Figure 9). Once it exits the midbrain, the oculomotor nerve runs anterolaterally within the interpeduncular cistern, piercing the arachnoid membranes delimiting the cistern itself. This segment of the nerve represents the lateral site of attachment of Liliequist’s membrane which, together with several arachnoid trabeculae of this region, contributes to the encirclement of the oculomotor nerve itself, thus constituting a well-defined arachnoid sheath that sends trabeculae to the uncus and to the tentorium (Figure 9).

The second segment, also known as the petroclinoid segment, extends from the posterior petroclinoid segment to the oculomotor porus, located in the central part of the oculomotor trigone [18]. The dural foramen of the oculomotor nerve is usually elliptical in shape as in the case observed in our series and is larger when the nerve crosses it in the majority of the cases (Figure 4, Figure 5, Figure 6, Figure 8a and Figure 9).

**Figure 11:**
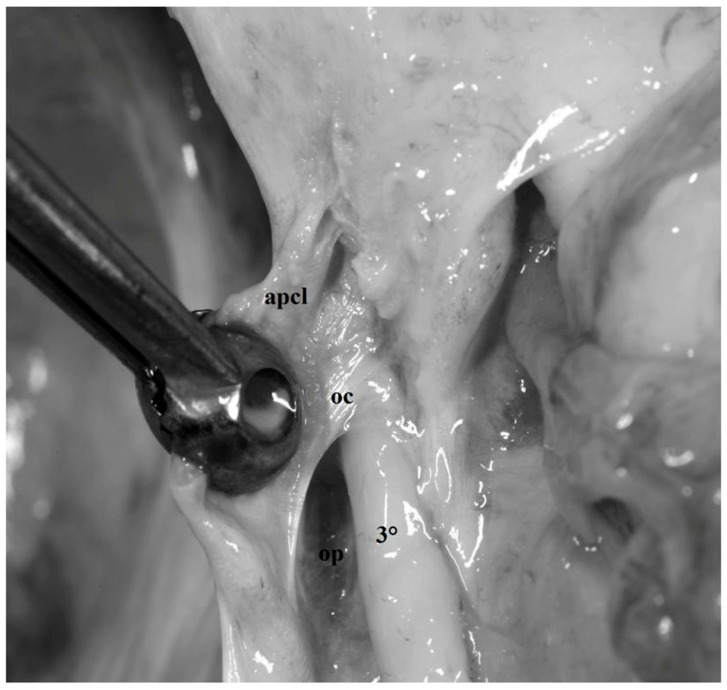
Fresh, non-formalin-fixed specimen, anatomic dissection, magnified view, left side. The anterior petroclinoid ligament (*apcl*) was cut and the lateral boundary of the oculomotor trigone was opened. This allowed us to visualize the trigonal portion of the oculomotor nerve (*3°*) after being passed through the elliptic oculomotor porus (*op*). Note how the trigonal portion of the nerve is localized within distinct cisternal space, also known with the name of oculomotor cistern (*oc*).

The trigonal portion begins where the oculomotor nerve pierces the oculomotor porus. In this portion, the nerve is encircled by its own cistern, also called the oculomotor cistern (Figure 11). The trigonal segment ends at the level of the inferior portion of the anterior clinoid process, where the nerve exits from its own cistern to enter the reticular dural layer constituting the lateral wall of the cavernous sinus. This point indicates the beginning of the cavernous segment which ends at the superior orbital fissure. In this portion, the oculomotor nerve is separated from the carotid artery by the above-mentioned carotid-oculomotor membrane (Figure 3).

### 3.12. Trochlear Nerve

The fourth cranial nerve may be divided into three distinct segments: cisternal, tentorial and cavernous. The first segment extends from the origin of the nerve at the brainstem below the quadrigeminal plate up to the point where it enters the tentorium (Figure 6). This segment passes within the quadrigeminal and ambient cistern and is related to the middle incisural space. In this portion, before entering the tentorium, the nerve usually comes into contact with the free edge of the tentorium and then disappears below the tentorium itself. This portion of the cisternal segment is also called infratentorial portion. In order to visualize this portion of the nerve during surgical procedures, the free edge of the tentorium must be lifted with a surgical hook (Figure 4, Figure 5 and Figure 6).

**Figure 12:**
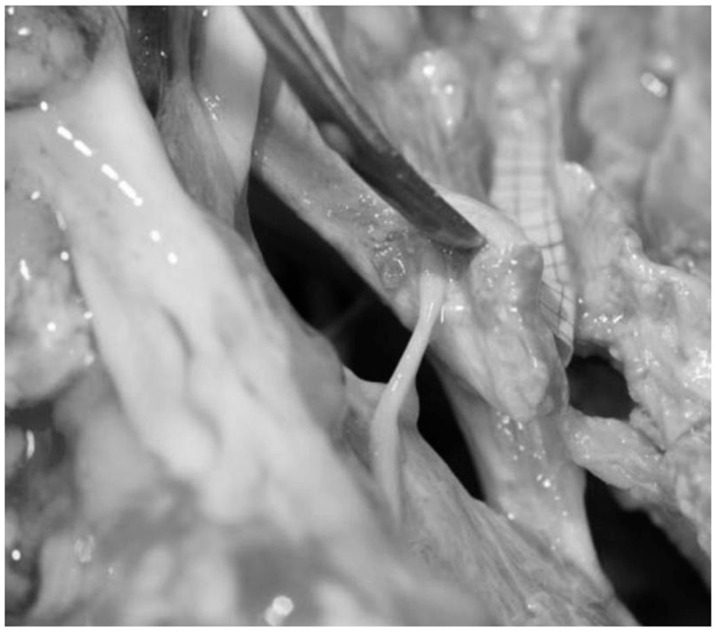
Fresh, non-formalin-fixed specimen, anatomic dissection, left side, magnified view. The tentorium was partially cut and lifted up through the dissector to show the point in which the infratentorial portion of the cisternal segment of the fourth cranial nerve penetrates into the tentorium.

The tentorial segment extends from the entrance of the trochlear nerve into the tentorium to the anterior petroclinoid fold, where the nerve enters the cavernous sinus (Figure 12) [29]. The nerve penetrates the tentorium below its free edge in a groove situated caudally to the postero-lateral margin of the oculomotor triangle, as described by Rhoton [11] and observed in our dissections. The dural opening for the visualization of the trochlear nerve is located in the angle between the anterior and posterior petroclinoid ligaments; in the vast majority of cases (63%), the diameter of the opening is equal to the diameter of the nerve (Figure 6) [29].

In the cavernous segment, the trochlear nerve enters the roof of the cavernous sinus in the postero-lateral apex of the oculomotor triangle, behind the entrance of the oculomotor nerve posterolaterally to the posterior clinoid. After entering the roof of the cavernous sinus, the nerve runs in the lateral wall of the cavernous sinus below the third cranial nerve. The cavernous segment ends at the superior orbital fissure.

### 3.13. Trigeminal Nerve

**Figure 13:**
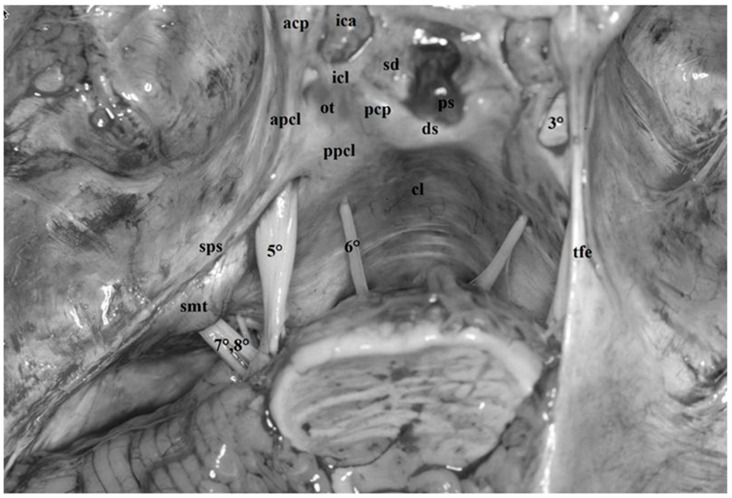
Fresh, non-formalin-fixed specimen, anatomic dissection, superior view. The cerebral hemispheres have been removed to show the skull base and its content. On the left side, the tentorium was cut and removed until the superior petrosal sinus (*sps*). The trigeminal root (*5°*) originates at the anterolateral margin of the pons and courses within the pre-pontine cistern toward the petrous apex, where it lies on the trigeminal impression. Here, the dura duplication of the tentorial edge’s anterior margin depicts a cavity called trigeminal porus. The porus represents the antrum through which the trigeminal nerve encircled by its own cistern enters the Meckel’s cave. Below the trigeminal nerve on the medial side, the abducens nerve piercing the clival dura (*cl*) is visible (*6°*) whereas slight laterally, the cochleo-vestibular-facial complex can be observed (*7°*, *8°*) partially covered by the suprameatal tubercle (*smt*) at the level of the acusticporus. At the petrous apex, the dura of the free edge of the tentorium splits into the anterior and posterior petroclinoid ligaments (*apcl*, *ppcl*) which, together with the interclinoid ligament (*icl*) between the anterior and posterior petroclinoid process (*acp*, *pcp*), delimits the oculomotor triangle (*ot*). The dura propria over the anterior clinoid process medially and posteriorly continues as diaphragm sella (*sd*) and clival dura after covering the dorsum sellae (*ds*). On the right side, the free edge of the tentorium (*tfe*) was left in place. (*ps*: pituitary stalk; *3°*: oculomotor nerve; *ica*: internal carotid artery).

The trigeminal nerve originates at the anterolateral margin of the pons and runs within the pre-pontine cistern toward the petrous apex, where it lies on the trigeminal impression. Here, the dural duplication of the anterior margin of the tentorial edge depicts a cavity called the trigeminal porus (Figure 6, Figure 7 and Figure 13). The trigeminal porus represents the antrum through which the trigeminal nerve, encircled by its own cistern, enters the cave of Meckel, located in the space between the periosteal and meningeal layer of the middle fossa (Figure 6, Figure 7 and Figure 13). The cave hosts the Gasserian Ganglion from which the three trigeminal roots origin.

### 3.14. Abducens Nerve

The abducens nerve ascends from the subtentorial part of the anterior incisural space. It originates from the pontomedullary sulcus, ascending in the prepontine cistern (which represents the only visible intracranial portion of the abducens nerve before perforating the dura mater covering the clivus), passing under the petrosphenoid ligament and finally entering the cavernous sinus (Figure 6, Figure 7 and Figure 13) [30].

## 4. Discussion

The anterior and the middle incisural spaces are regions of exceptional anatomical and surgical interest [1,2,3]. The term “incisural space” was introduced by Rothon and it describes the region located between the free edge of the tentorium and the upper brainstem. As described above, the anterior incisural space is situated anteriorly to the brainstem, whereas the middle incisural space is lateral to it.

These regions are characterized by complex relationships between the bony structures of the skull base, dura mater, arachnoid membranes, cisternal spaces, cranial nerves and vascular structures [1,2,3,7,8,11,23,24,31,32].

Although several studies focusing on this topic have already been performed, previous published papers analyzed formalin-fixed specimens [1,3,5,7,9,11].

Several studies have reported that formalin interacts with the arachnoid membranes by altering their morphological structure [17,26]. This interaction is responsible for alterations of the results of studies performed during such dissections [14,29].

In this study, we used fresh, non-formalin-fixed specimens. This opportunity made it possible to improve our understanding and description of the anatomical relationships between the dural folds and the arachnoid membranes of this region.

The goal of our dissections was the region of the skull base known as the oculomotor triangle, characterized by dural folds running between the petrous apex and the anterior and posterior clinoid processes [1,2,3,8]. In our study, the three main components of this triangle were clearly identified in all the dissections performed. The anterior and posterior petro-clinoid ligaments, running between the petrous apex and the anterior and posterior petro-clinoid processes, respectively, derive from a duplication of the free edge of the tentorium at the petrous apex. The third component of the triangle is represented by the interclinoid ligament formed by a thickening of the dura propria extending between the anterior and posterior clinoid processes (Figure 4, Figure 5 and Figure 6).

The petroclinoid portion of the oculomotor nerve penetrates the triangle in its central part passing through an elliptical opening known as the oculomotor porus [1,9] (Figure 4 and Figure 5). Incision of the anterior petroclinoid ligament and opening of the oculomotor porus reveals, in fresh specimens, the arachnoid membrane encircling the oculomotor nerve and delimiting its own cisternal space [26,31] (Figure 11). The thick dural layer composing the oculomotor triangle and covering the superior surface of the anterior clinoid process is called “meningeal dura” or “dura propria” and is continuous with the dura of the falciform ligament and diaphragma sellae. Moreover, on the medial side, the dura propria encircles the ICA, thus constituting the distal dural ring which represents the superior limit of the clinoid segment of the ICA itself [1,2,3,4,5,8] (Figure 3).

Intradural removal of the anterior clinoid process exposes a second triangular space called the carotid triangle, which is mainly constituted by a second, thinner layer of dura known as “periosteal” or “reticular” dura [1,2,3,7]. This layer constitutes the so-called carotid-oculomotor membrane which, encircling the internal carotid artery, forms the proximal dural ring and the carotid collar [1,2,3,8]. The first structure defines the origin of the clinoid segment of the ICA, whereas the collar adheres to the ICA itself in its clinoid portion thus forming a pouch on the medial side, also known as carotid cave, which is a common site of formation of intracranial aneurysms [1,2,3,4,5,8] (Figure 3).

In our dissections, it was possible to appreciate the arachnoid sheets dividing the chiasmatic one from the carotid cistern and running posteriorly. This course contributes to the formation of the anterolateral portion of the cone-shaped arachnoid ring, delimiting the pituitary stalk cistern [8,16] (Figure 8a–c and Figure 9). At the level of the dorsum sellae, the merits of our dissection technique appear to be even more evident. In fact, in all our dissections performed on fresh specimens, it was possible to clearly delineate the anatomy of the membrane of Liliequist, identifying both its mesencephalic and the diencephalic portions. To our knowledge, no study has previously been published in the literature identifying both components of this membrane as clearly as we did in the present study. In particular, we observed how the diencephalic portion of Liliequist’s membrane is attached to mammillary bodies and how the intracisternal portion of the third cranial nerve represents the pillar to which the mesencephalic portion of the membrane is attached laterally, thus incompletely separating the prepontine from the interpeduncular cisterns [8,16]. (Figure 8a–c, Figure 9 and Figure 10). Moreover, it was possible to identify the trabeculae of the membrane itself, running from the dorsum sellae towards the posterior surface of the pituitary stalk completing the arachnoid collar encircling the stalk itself and delimiting its cisternal space [16] (Figure 9 and Figure 10).

The adjacent structures identified in our dissections were the petroclinoid ligaments, running below the posterior petroclinoid ligament and constituting the roof of Dorello’s canal through which the abducens nerve penetrates the cavernous sinus (Figure 6 and Figure 7), the fourth cranial nerve in its cisternal and tentorial segments (Figure 4, Figure 5, Figure 6 and Figure 12) and the trigeminal root passing from the prepontine cistern to Meckel’s cave through the trigeminal porus at the petrous apex [2,3,9,11] (Figure 6, Figure 7 and Figure 13).

**Figure 14:**
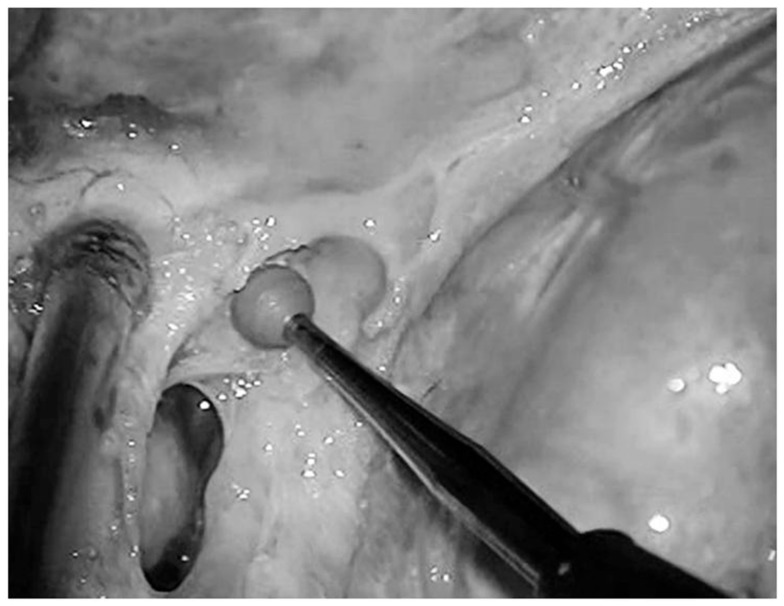
Fresh, non-formalin-fixed specimen, anatomic dissection, anterolateral view of the right side. The cerebral hemispheres have been removed to show the skull base. The intradural clinoidectomy is being performed. After the incision of the meningeal layer and the dissection of the periosteal layer from the inferior surface of the clinoid the clinoid itself is detached from the skull base through the drilling of its three insertion points: lesser wing of the sphenoid, planum sphenoidale and optic strut.

**Figure 15:**
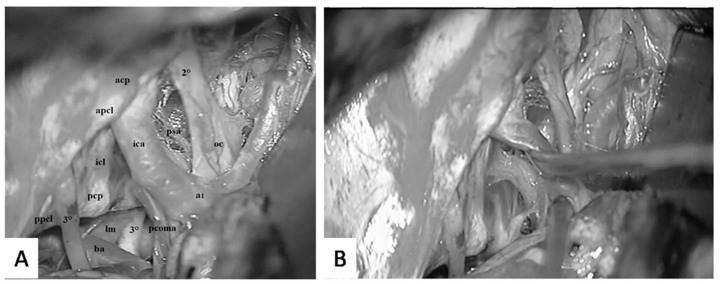
(**A**) Intraoperative photograph, pre-temporal view, left side. (**A**) Left temporal polectomy was performed for the removal of a glioblastoma cerebri. The anterior incisural space was exposed. The component of the oculomotor triangle can be clearly observed (*apcl*: anterior petroclinoid ligament; *ppcl*: posterior petroclinoid ligament; *icl*: interclinoid ligament). In the central part of the triangle, the oculomotor nerve (*3°*) enters the oculomotor porus. The oculomotor nerve can be followed posteriorly in its cisternal within the interpeduncular cistern until it reaches its origin at the brainstem below the posterior cerebral artery. On the left side, the arachnoid membranes including the Lilequist’s membrane were dissected. Conversely, on the right side, the mesencephalic portion of the Lilequist’s membrane attaching on the contralateral third cranial nerve (*3°*) can be identified. Anteriorly, on the left side, between the ICA (*ica*) and the optic nerve (*2°*), trabeculae coming from the medial carotid membrane and reflecting over the pituitary stalk can be observed (*psa*) (*a1*: anterior cerebral artery; *oc*: optic chiasm; *acha*: anterior choroideal artery). (**B**) After the dissection of the arachnoid membranes of the anterior incisural space has been completed, the ICA can be easily mobilized. In this photo, the dissector has been used to show the pituitary stalk dislocating the ICA antero-medially.

**Figure 16:**
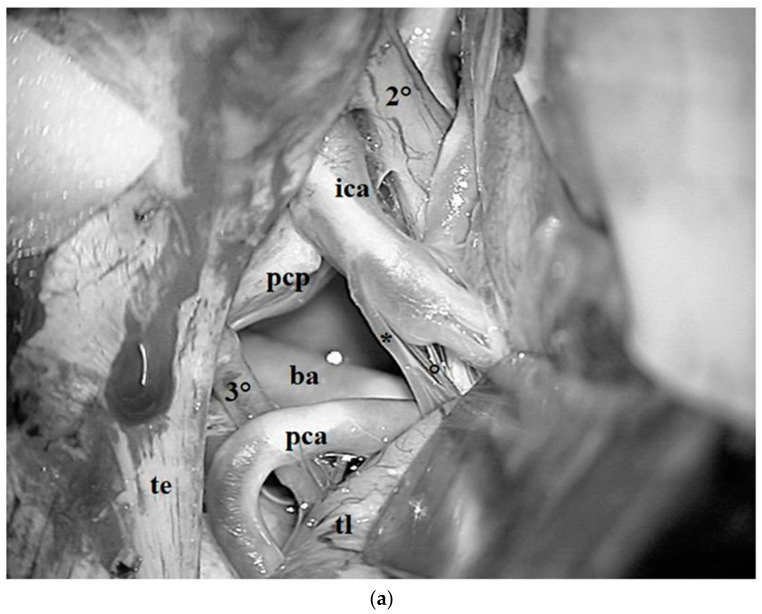

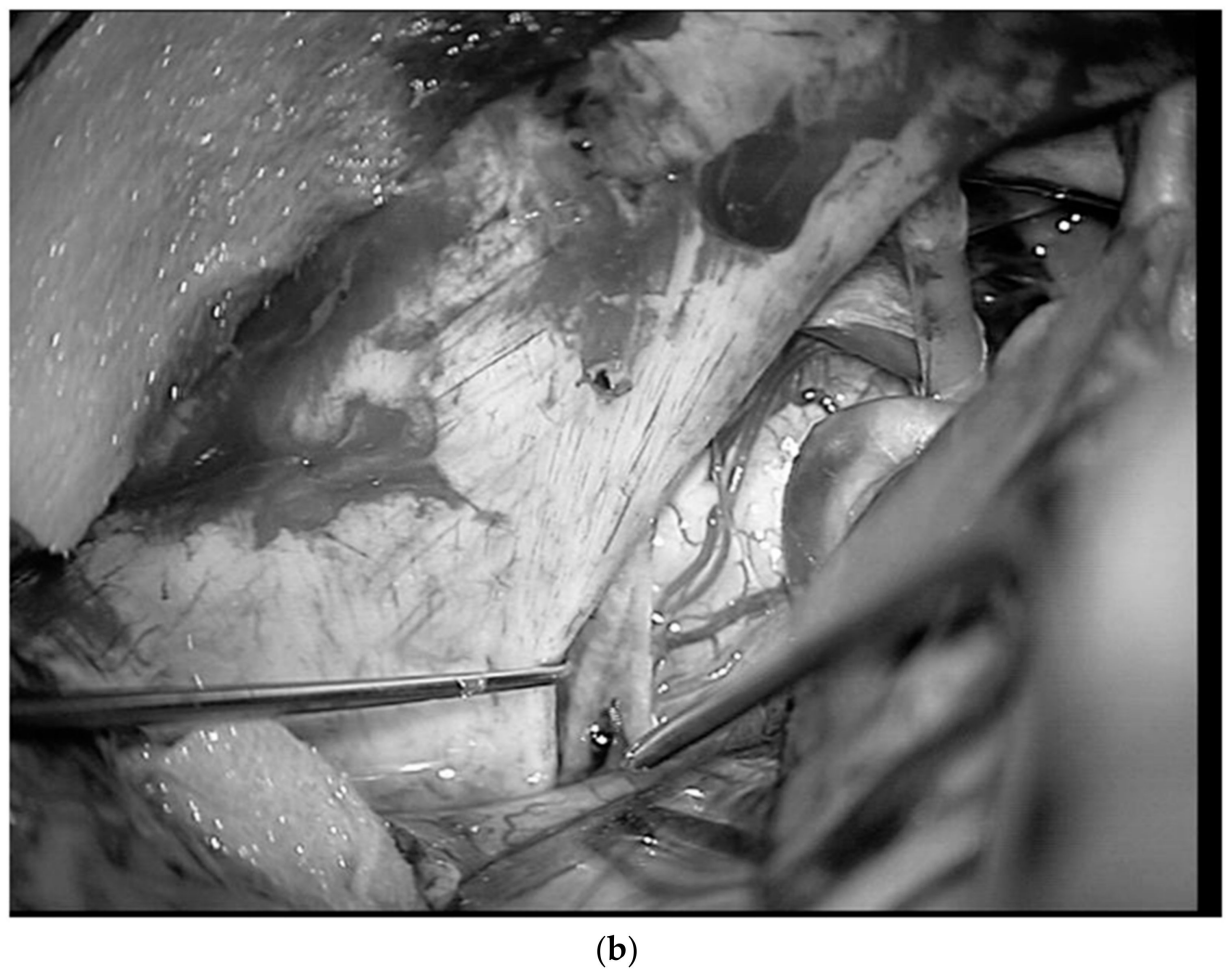
(**a**) Intraoperative photograph, subtemporal view left side. The left temporal lobe (*tl*) has been lifted up and the middle incisural space has been exposed. A more lateral route increases the operative view of the interpeduncular cistern. The third cranial nerve (*3°*) can be followed until its origin below the posterior cerebral artery (*pca*). Medially, both the anterior posterior communicating artery (*) and its perforators constituting the pre-mammillary artery (*°*) can be identified (*te*: tentorial edge; *ica*: ICA; pcp: posterior clinoid process; *2°*: optic nerve). (**b**) The tentorial edge (*te*) has been lifted up through the use of a microsurgical hook. Below the microsurgical hook, the fourth cranial nerve running within the ambient cistern can be observed.

**Figure 17:**
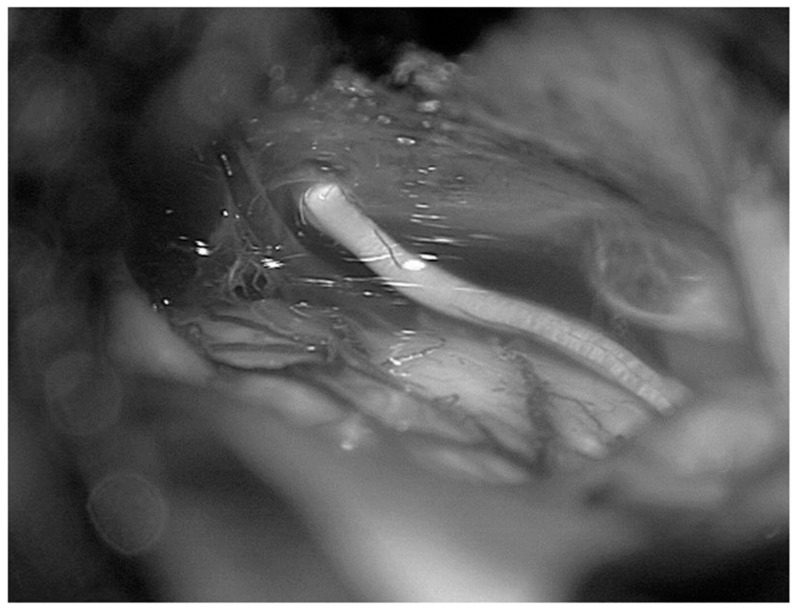
Intraoperative photograph. A right temporo suboccipital approach has been performed and the tentorium has been opened. The infratentorial compartment of the middle incisural space has been exposed and the sixth cranial nerve entering the Dorello’s channel can be observed.

Thorough knowledge of the anterior and middle incisural spaces with their related bony, dural, arachnoid and neurovascular structures is mandatory in neurosurgical practice. 21 In fact, these regions are a common site of origin for vascular and neoplastic pathologies and are among the surgical regions most frequently exposed during the most widely used neurosurgical approaches, such as the pterional, the pretemporal and the subtemporal routes [5,6,8,10,15,16,23,24,33] (Figure 14, Figure 15, Figure 16 and Figure 17).

Although the above-mentioned osteodural structures play a role in upholding the anatomical relationships between the neurovascular components of these regions, their presence also represents an obstacle to the surgical exposure and complete visualization of those neurovascular structures with which they interact [1,2,3,7,8,11].

As a consequence, the complete or partial removal of the osteodural structures as explored by our dissections represents the key for unlocking such surgical approaches by widening surgical access [5,6,8,10,15,16].

Intradural or extradural removal of the anterior clinoid process is commonly performed in neurosurgical practice [6,8,15] (Figure 14). This maneuver allows exposure of the clinoid segment of the ICA ensuring proximal control in the management of paraclinoid aneurysms, including “carotid cave”, hypophyseal and carotid-ophtalmic aneurysms [6,8,15]. Moreover, removal of the anterior clinoid process makes it possible to unroof the optic canal and to remove tumors spreading within the canal itself, such as meningiomas and craniopharyngiomas [6,8,15,16,26]. With reference to craniopharyngiomas, a thorough knowledge of the arachnoid membranes around the pituitary stalk has been demonstrated to be pivotal during surgical removal of these lesions, since the relationships between the lesion, the basal arachnoid membrane and the components of the medial carotid and of Liliequist’s membrane over the infundibulum influence the possibility of identifying an anatomical plane between the tumor, the pituitary stalk, the optic nerve/chiasm and the arterial components of the region [16,26].

Following an anterior clinoidectomy, further surgical space may be obtained by cutting the dura mater of the distal dural ring and opening the carotid-oculomotor membrane, accompanied by a meticulous dissection of the arachnoid adhesions of this region [8]. These maneuvers are of particular importance in the pre-temporal approach, since they allow an extensive exposure of the oculomotor nerve from the trigonal to the cisternal portion and ensure a wide visualization of the interpeduncular cistern [6,8] (Figure 15a,b). This last aspect demonstrates how a well-performed pre-temporal approach, accompanied by an extensive mobilization of dural and arachnoid membranes, may reasonably be considered the main surgical solution in the management of high basilar bifurcation aneurysms and of pathological conditions located in the upper ventral brainstem [6,8,15]. Moreover, the possibility of simultaneously performing a posterior clinodectomy and closure of the posterior communicating artery at the P1–P2 junction, makes the pre-temporal approach a valid surgical alternative with respect to the subtemporal one, even in case of low-lying basilar bifurcation aneurysms [6,8,10,15,21,23].

The above-mentioned subtemporal approach is a versatile approach mainly performed for exposure of the middle incisural space [10,15] (Figure 16a,b). Using this approach, the ambient and the interpeduncular cisterns may be widely exposed [10,15]. In neurosurgical practice, this approach is mainly used for the management of low-lying basilar bifurcation aneurysms, meningiomas of the free margin of the tentorium and lesions involving the lateral portion of the mesencephalon and the upper lateral pons at the trigeminal root origin. This approach offers the possibility of making an incision on the tentorial edge after visualization of the entry point of the fourth cranial nerve together with the dissection of the arachnoid of Liliequist’s membrane, widening the surgical view and exposing both the supratentorial and infratentorial portion of the upper brainstem [10,15] (Figure 16a,b and Figure 17).

To sum up, our study, performed on both fresh non-formalin-fixed as well as on formalin-fixed specimens, concretely demonstrated the usefulness of an anatomical study applied to neurosurgical practice. A systematic approach based on the stepwise analysis of the dural, bony and neurovascular structures as well as, in the case of dissections performed on fresh cadavers, arachnoid membranes and cisterns, is able to provide neurosurgeons with the indispensable anatomical basis necessary for successfully managing a variety of pathologies involving the anterior and middle incisural spaces [34].

## 5. Conclusions

The anterior and middle incisural spaces are regions of exceptional anatomical and neurosurgical interest characterized by complex relationships between bony, dural neurovascular and arachnoid structures. The use of fresh cadaveric specimens, thus avoiding the morphologic alterations determined by the fixation process, permitted us to clearly understand how the arachnoid folds and cisterns of the region interact with the petroclinoid folds of this area and with the skeletal components of the sphenoid and petrous bone [21]. A thorough knowledge of the anatomy of this complex region has a concrete application in neurosurgical practice since the anterior and middle incisural spaces are common sites of insurgence of vascular and neoplastic pathologies and are among the most frequently exposed surgical regions in widely used neurosurgical approaches such as the pterional, pretemporal and subtemporal routes.

## Data Availability

Not applicable.
